# L-tyrosine inhibits the formation of amyloid fibers of human lysozyme at physiological pH and temperature

**DOI:** 10.1007/s00726-025-03445-6

**Published:** 2025-02-16

**Authors:** Santos López, Arturo Rojo-Domínguez, Roxana López-Simeon, Alejandro Sosa-Peinado, Hugo Nájera

**Affiliations:** 1 Posgrado en Ciencias Naturales E Ingeniería, Universidad Autónoma Metropolitana—Cuajimalpa, Alcaldía Cuajimalpa de Morelos, Av. Vasco de Quiroga 4871, Colonia Santa Fe Cuajimalpa, Ciudad de México, 05348 México; 2 Departamento de Ciencias Naturales, Universidad Autónoma Metropolitana—Cuajimalpa, Alcaldía Cuajimalpa de Morelos, Av. Vasco de Quiroga 4871, Colonia Santa Fe Cuajimalpa, 05348 Ciudad de Mexico, Mexico; 3 Laboratorio de Biosensores y Modelaje Molecular, Departamento de Bioquímica, Facultad de Medicina, Universidad Nacional Autónoma de México, 04510 Ciudad de Mexico, Mexico

**Keywords:** Lysozyme, L-tyrosine, Inhibition, Docking

## Abstract

Amyloid fibers are implicated in numerous diseases, making their study crucial for identifying effective therapeutic compounds. This research highlights the ability of L-tyrosine to inhibit the formation of amyloid fibers in human lysozyme. At a 1:1 molar ratio under physiological conditions (pH 7.4, 37 °C), L-tyrosine significantly reduces amyloid fiber formation, as evidenced by a decrease in thioflavin T fluorescence. Differential scanning calorimetry (DSC) shows a major energy requirement for temperature denaturation when the lysozyme is in the presence of L-tyrosine. Additionally, chemical denaturation experiments reveal a shift in the intrinsic fluorescence spectrum of lysozyme in the presence of L-tyrosine, indicating a direct interaction. Computational docking studies with Molecular Operating Environment (MOE) further confirm that L-tyrosine binds effectively, exhibiting similar binding energies to those of the natural substrate. This study underscores L-tyrosine’s potential as a strong inhibitor of amyloid fiber formation, demonstrating its stabilizing effect on lysozyme and its promise in therapeutic applications.

## Introduction

Amyloids are deposits both extracellular and intracellular of insoluble fibrillar proteins characterized by predominantly β-sheet secondary structures. Initially identified in a group of disorders known as amyloidosis, their presence has since been linked to a broad spectrum of diseases, including Parkinson’s, Alzheimer’s, and systemic amyloidosis, among others (Eisenberg and Jucker 2012). Given their significant role in these conditions, studying compounds capable of inhibiting amyloid formation is crucial (Walsh et al. 2002). While numerous compounds have been investigated, only a few exhibit the desired inhibitory effects, and even fewer are suitable for therapeutic use in humans. Natural compounds, including certain phenols and amino acids, show promise in this regard (Shoval et al. 2007). In this way, some amino acids were used as templates for deploying different inhibitors of fibers; for example, non-natural amino acids were used to inhibit the amyloid fibers (Sievers et al. 2011), and others were combined with silver nanoparticles to inhibit Insulin fibers (Bera et al. 2023). L-tyr was used as a base for researching new compounds as amyloid-beta aggregation inhibitors (Xu et al. 2023). On the other hand, it was used to find an attenuator of the aggregation and neurotoxicity of amyloid peptides (Xu et al. 2019).

Amyloid fibril formation involves a nucleation-dependent self-assembly process. This process begins with a lag phase during which the fibril nucleus forms, followed by an exponential growth phase where fibrils assemble. Structurally, amyloids consist of two or more protofilaments –unbranched proteinaceous fibers approximately 5–15 nm in width and extending into the micron range. A defining feature of amyloid fibrils is their core cross-β structure, with protofilament subunits dependent on their precursor proteins (Hassan et al. 2022).

In vitro studies using peptides and proteins associated with specific diseases have provided valuable insights into the physicochemical properties underlying amyloid aggregation, offering clues to the mechanisms at play in vivo (Marcon et al. 2005).

Lysozyme, a glucoside hydrolase in the human body (Parry et al. 1969), is a well-established model for studying amyloid fiber formation due to its well-characterized structure and behavior. It features two distinct structural domains, α- and β-domains (Swaminathan et al. 2011). Specific mutations in the β-domain, such as D67H and I56T, are linked to amyloid fiber formation and systemic amyloidosis (Pepys et al. 1993).

This study explores the potential of L-tyrosine to inhibit amyloid fiber formation in human lysozyme under physiological conditions (pH 7.4, 37 °C). While various amino acids have been tested in different models –for instance, arginine has been shown to inhibit β-casein (Wang et al. 2022), evaluating amino acids like L-tyrosine for their efficacy in similar contexts is crucial. Previous research has examined numerous compounds in the lysozyme model, including small molecules like rutin, curcumin, and quercitin, as well as macromolecules and polymers such as bovine serum albumin (BSA), poly (norbornene gluconamide), and pectin, among others. However, many of these compounds are only effective at non-physiological pH levels, with few maintaining activity under physiological conditions (Chen et al. 2022).

Our research highlights a significant finding: certain short peptides, or amino acids, including L-tyrosine, exhibit a unique ability to inhibit amyloid fiber (Neddenriep et al. 2011). This discovery offers promising potential for identifying compounds with desirable characteristics for amyloid inhibition. Notably, L-tyrosine stands out as a natural compound with the ability to inhibit amyloid formation at physiological pH, making it a particularly valuable candidate for further study.

## Materials and methods

### Materials

Thioflavin T (ThT), human lysozyme, urea, L-tyrosine (L-tyr), and 8 anilinonaphthalene-1-sulfonic acid (ANS) were sourced from Sigma-Aldrich.

### Amyloid fiber formation

Human lysozyme was prepared in a stock solution at a concentration of 50 mg mL^−1^ in 20 mM potassium phosphate buffer, pH 7.44, which was used for all experiments. ThT was prepared as a 10 mM stock solution in absolute ethanol. Fibrillogenesis was assessed using a Costar 96-well plate and monitored by ThT fluorescence in a plate reader (TECAN Infinite M1000Pro). The final concentrations in each well were 1725 μM lysozyme and 66 μM ThT, all in the potassium phosphate buffer. Samples were excited at 450 nm, with emission detected at 490 nm. Kinetics were measured every 10 min for 16 h at 37 °C.

### Fibrillogenesis in the presence of L-tyr

L-tyrosine was prepared as a 2.5 mM stock solution. Inhibition assays were conducted using a 1:1 molar ratio of lysozyme to L-tyr. Fibrillogenesis was monitored using ThT fluorescence in a plate reader (TECAN Infinite M1000Pro) under the conditions described in Sect. "Amyloid fiber formation". all in the potassium phosphate buffer and with 1:1 molar ratio. Excitation was at 450 nm, with emission measured at 490 nm. Kinetics were recorded every 10 min for 25 h at 37 °C.

### ANS fluorescence

ANS was prepared as a 5 mM stock solution in potassium phosphate buffer. Two experimental conditions were evaluated: 1) amyloid fiber formation with lysozyme alone (at a final concentration of 25 mg mL^−1^), and 2) in the presence of L-tyr at a 1:1 molar ratio with lysozyme. ANS was used at a final concentration of 20 μM for both conditions. ANS fluorescence was monitored using a plate reader (TECAN Infinite M1000Pro). Samples were excited at 365 nm, with emission detected at 450 nm. Kinetics were recorded every 10 min for 16 h at 37 °C.

### Circular dichroism (CD)

CD spectra were recorded and incubated at 37 °C for native, amyloid fibers of human lysozyme, and the result of the fibrillogenesis in the presence of L-tyr using a Jasco J-815 CD spectropolarimeter (Tokyo, Japan) equipped with a cell holder, with a Peltier temperature control over the range of 260 to 190 nm. All CD spectra reported are the average of three consecutive scans. CD spectra data were analyzed using the “BESTSEL” program.

### Heat denaturation

Heat denaturation experiments were conducted using a Spectrofluorometer Chronos BH equipped with a steady-state fluorescence package and a 1 cm pathlength quartz cuvette. The intrinsic fluorescence of tryptophan was used as the reporter. Samples were excited at 295 nm, and emission was collected at 334 nm over 65 min, with a temperature ramp rate of 1 °C min^−1^, starting at 25 °C and ending at 90 °C. Lysozyme stability was tested at a concentration of 12.5 mg mL^−1^, both in the absence and presence of L-tyr at a 1:1 molar ratio.

Data were collected and processed using Origin 2016. The initial analysis involved fitting the data to a Boltzmann model for the initial phase:

1 $$ y\, = \,A_{2} \, + \,\left( {A_{1} - A_{2} } \right)/\left( {1\, + \,\exp \left( {\left( {x - x_{0} } \right)/dx} \right)} \right) $$

A_1_ represents the initial value, A_2_ is the final value, x_0_ is the center of the transition, and dx is the time constant.

For the latter part of the experiment, maximum fluorescence values were used for comparison, as reliable fits were not obtained for all data sets.

### Differential scanning calorimetry (DSC)

Samples were analyzed using a MicroCal VP-Capillary DSC. Two experimental conditions were investigated: 1) lysozyme at a concentration of 0.02 mM in phosphate buffer at pH 7.44, and 2) lysozyme with L-tyr at a 1:1 molar ratio, also in phosphate buffer. Thermal analysis was performed with a heating rate of 1 °C min^−1^ over a 15–90 °C temperature range. Data were collected and processed using Origin software with the DSC package. To ensure that only the heat transition due to protein conformational changes contributed to the thermal differences between the sample cell and the reference cell, a baseline thermogram was obtained by loading buffer into both the sample and reference cells. This baseline was subtracted from the sample thermogram during data analysis. The transition temperature, Tm, and the peak maximum calorimetric enthalpy, ΔH, were determined from the area under the transition profile with a stepped baseline.

### Urea denaturation

Denaturation experiments were conducted using a Spectrofluorometer Chronos BH equipped with a steady-state fluorescence package and a 1 cm pathlength quartz cuvette. Intrinsic tryptophan fluorescence was used as the reporter. Samples were excited at 295 nm, and emission was collected at 334 nm over a period of two hours. The final concentrations were 6.5 M for urea, 12.5 mg mL^−1^ for lysozyme, and a 1:1 molar ratio of L-tyr to lysozyme. Data were collected and processed using Origin 2016 and fitted to the BiDoseResp model.

2 $$y={\text{A}}_{1}+({\text{A}}_{2}-{\text{A}}_{1})[\frac{\text{p}}{1+{10}^{\left(\text{LOGx}01-\text{x}\right)\text{h}1}}+\frac{1-\text{p}}{1+{10}^{\left(\text{LOGx}02-\text{x}\right)\text{h}2}}]$$

Where A_1_ = Bottom value, A_2_ = Top value, LOGx01 = 1st EC_50_, LOGx02 = 2nd EC50, h1 = slope1, h_2_ = slope2, p = proportion-

### Scanning electron microscopy SEM

The microscopy was performed using a Hitachi TM3030Plus. For sample preparation, a sample drop was placed on a glass coverslip and allowed to dry for 24 h. The coverslip with the sample was then mounted onto the microscope stage using carbon tape. All samples were analyzed after 16 h of incubation.

### Docking

Computational docking studies were conducted using the Molecular Operating Environment (MOE. Version 2007). The structure of human lysozyme was obtained from PDB (ID 3FE0) and analyzed with the same program. The ligand (L-tyr) was obtained from PubChem (CID 6075). The CHARMM27 force field was applied to the enzyme, and the MMFF94x force field to the ligand, for partial charge assignation. The entire protein surface was used to define the interaction site and pose search with the Alpha tringle method. Up to 15,000 potential poses were assayed in each computational experiment. The scoring function employed was London dG, and the top 30 results were refined using this function to optimize the poses. Interaction maps were generated using MOE’s analysis tools.

## Results and discussion

### Amyloid fibrils at physiological pH and temperature

**Fig. 1:**
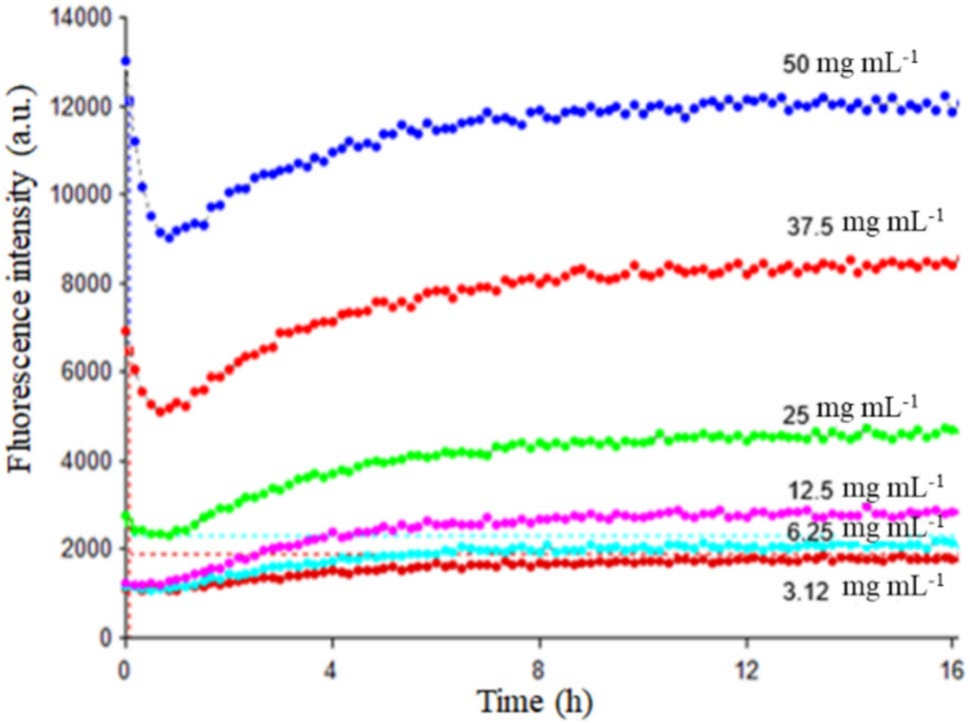
Fibrillogenesis at different lysozyme concentrations: 3.12, 6.25, 12.5, 25, 37.5, and 50 mg mL^−1^ (dark red, cyan, magenta, green, red, and blue, respectively). ThT fluorescence was used as the reporter

In this study, we investigated the formation of amyloid fibers at physiological pH and temperature, both in the presence and absence of L-tyr. The formation of amyloid fibrils was monitored using ThT fluorescence, a widely used reporter that increases fluorescence emission upon binding to amyloid fibers (Biancalana and Koide 2010). Amyloid fibril formation at physiological pH and temperature was assessed at various protein concentrations, ranging from 3.12 to 50 mg mL^−1^, over 16 h. The effect occurs because the concentration is very high compared with the physiological concentration (between 7 to 13 mg/L) (Hankiewicz and Swierczek 1974; Pankratov et al. 2023); in this way, not all the variables are physical. As shown in Fig. 1, the ThT signal increased with higher lysozyme concentrations, indicating more robust fibril formation (Khurana et al. 2005; Biancalana and Koide 2010).

Among the tested concentrations, 25 mg mL^−1^ lysozyme yielded the most reliable results. Higher concentrations, specifically 37.5 and 50 mg mL^−1^, were less consistent due to difficulties in achieving homogeneous solutions. This means the presence of precipitates in the solution and the appearance of a coil at the onset in the ThT measurements, which were attributed to the high protein concentration.

Figure 1 illustrates that only a high concentration is enough to form amyloid fibrils; additionally, they are fully formed within 8 h at 25 mg mL^−1^ lysozyme concentration and 37 °C, although the experiment was conducted for 16 h. Consequently, 25 mg mL^−1^ was selected for further studies on inhibition with L-tyrosine. Microscopic analysis, as shown in Fig. 8, confirmed the presence of amyloid fibers, demonstrating that human lysozyme can form these structures under physiological conditions of pH 7.44 and 37 °C but only at elevated protein concentrations.

### Inhibitory effect of L-tyr in fibrillogenesis of human lysozyme

**Fig. 2:**
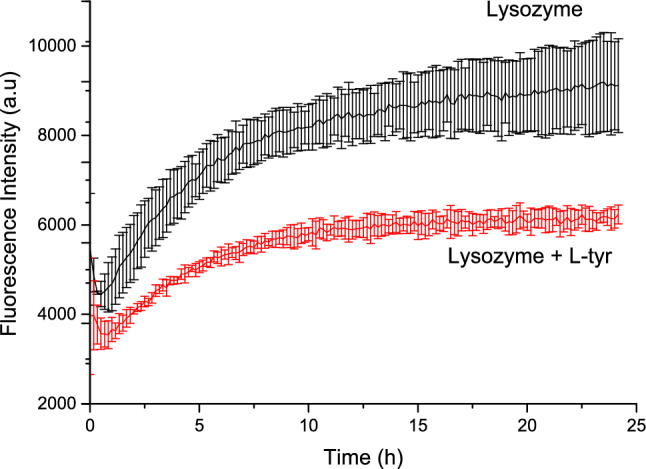
Inhibition of human lysozyme amyloid fibers by L-tyrosine

When combined with other molecules and proteins, certain amino acids, have been shown to act as inhibitors of fibrillogenesis (Dubey et al. 2015). In this study, L-tyr was investigated as an inhibitor, and the results are depicted in Fig. 2. The data reveal a significant reduction in fluorescence intensity. This decrease indicates a reduction in β-sheet content, which is fundamental to the structure of amyloid fibers, as ThT specifically interacts with these β-sheet structures (Biancalana and Koide 2010; Toyama and Weissman 2011). These findings align with existing literature, which suggests that polyphenols typically exhibit fibril inhibition properties (Ngoungoure et al. 2015; Bloch et al. 2020).

The results demonstrate a notable inhibitory effect of L-tyrosine, highlighting its potential significance in the field. This observation supports the notion that aromatic amino acids, rather than being essential for amyloid fiber formation, can disrupt the process (Stanković et al. 2020). Indeed, many peptides that inhibit amyloid fibrillogenesis include aromatic amino acids in their structure (Mitra and Sarkar 2020).

Notably, the inhibition was effective at a 1:1 molar ratio (lysozyme to L-tyr), emphasizing its potential as a preventive agent. The inhibition effect was evident early in the fibrillogenesis process, suggesting a promising preventive role. Furthermore, as shown in Fig. 8, Panel D, the inhibition resulted in the formation of species distinct from amyloid fibers, although these are not the native lysozyme form. These species are likely oligomers, which retain the ability to bind ThT, indicating the presence of β-sheet structures with a specific affinity for the dye. This characteristic is consistent with other oligomers formed under low pH conditions (Malisauskas et al. 2006). It is important, considering that earlier studies have shown that L-tyr can self-assemble and act as a seed for other proteins, promoting fibrillation; these studies differ from the present results because the methodology is different; they heat the solution of L-tyr to 90 °C and then gradually cooled to form the fibers (Zaguri et al. 2018), and in the other they mimic the physiological conditions as us but changes the time of the experiment (Anand et al. 2018), the fibers appears after 25 h of incubation, contrasting the results shown in Fig. 1, where the inhibition process occurs in the first 2 h this suggests that L-tyr interact first with the protein than with herself.

### ANS fluorescence

Hydrophobicity was assessed using ANS, which interacts with hydrophobic regions of proteins or their aggregates (Stryer 1965).

**Fig. 3:**
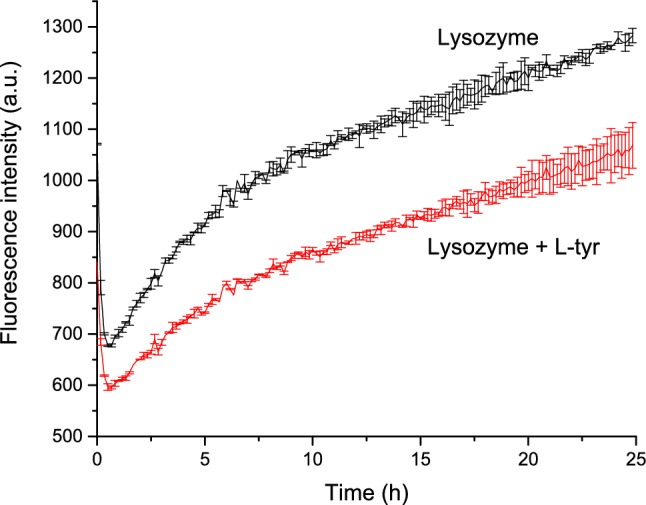
Effect of L-tyr in the ANS fluorescence kinetics of amyloid fiber formation. Lysozyme in the absence of L-tyr (black) and in the presence of L-tyr (red)

Figure 3 illustrates that ANS fluorescence intensity is significantly higher in the absence of L-tyr, indicating that fewer hydrophobic regions are exposed when the L-tyr is present. This suggests a difference in the process and final stages between lysozyme alone and lysozyme with L-tyrosine. This finding aligns with the results obtained from thioflavin T assays, where the fluorescence intensity of the dye correlates directly with the presence of amyloid fibers (Sulatsky et al. 2020) L-tyr indicates that the compound’s effect manifests early in the fibrillogenesis process. L-tyr appears to stabilize the protein, preventing the formation of additional β-sheets of amyloid fibers and thereby exposing fewer hydrophobic areas.

These observations suggest that L-tyr interrupts the fibrillogenesis process at its onset, likely by stabilizing the protein and inhibiting the nucleation of amyloid fibers. Consequently, the intermediates formed –likely oligomers– exhibit β-sheeted structures but expose fewer hydrophobic regions than mature amyloid fibers.

### CD

**Fig. 4:**
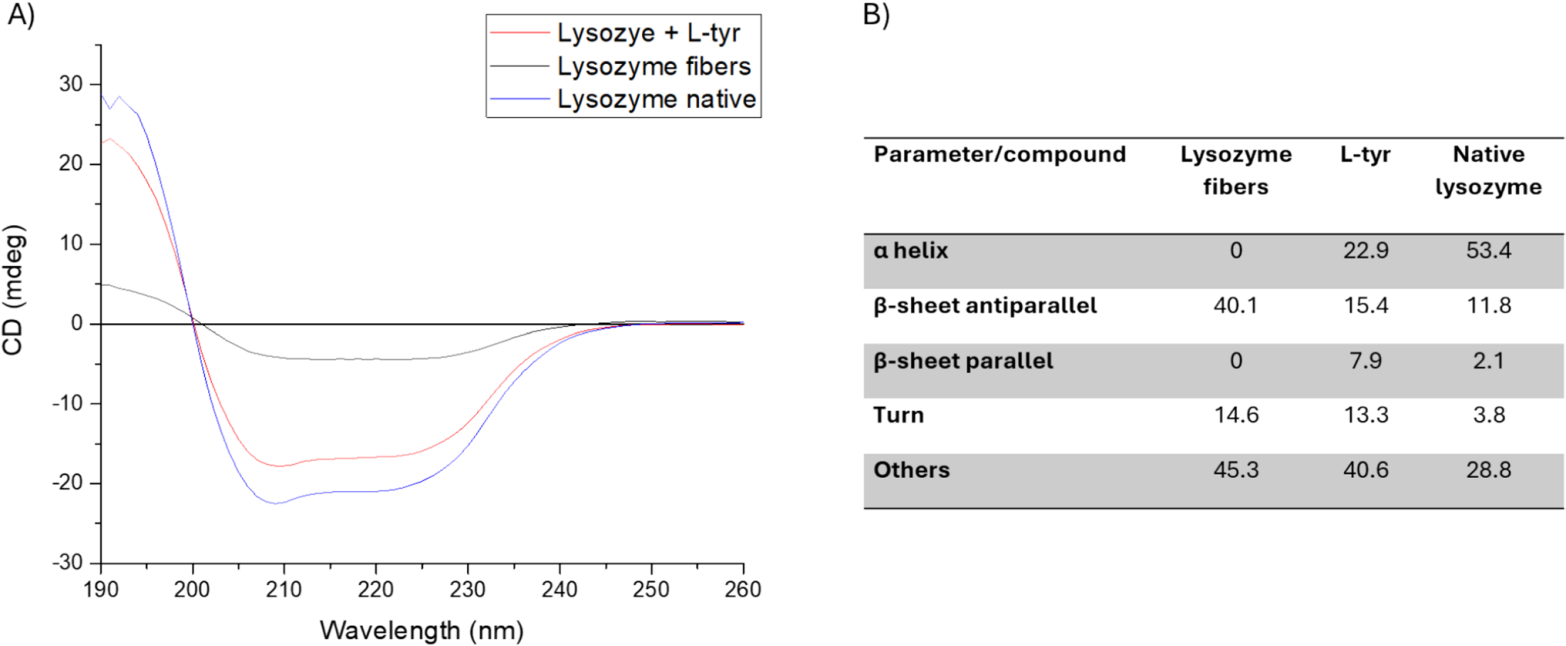
**A** Far-UV CD spectral changes of lysozyme during fibrillation and the final structure after the effect of L-tyr in the fibrillogenesis. **B** CD analysis for lysozyme, lysozyme fibers, and lysozyme in the presence of L-tyr

Far UV-CD is a widely used spectroscopic technique to analyze the secondary structural changes in proteins. In the case of lysozyme fibrillation, conformational changes can also be monitored using this technique (Das et al. 2024). The transition from the native form to amyloid fibrils is characterized by a shift in structure to a β-sheet conformation (Serpell 2000). Figure 4 shows the structural changes in lysozyme when modified to fibers and when L-tyr is present. It can be observed for native lysozyme (blue line), a negative peak distinguishes native human lysozyme at 208 nm and a negative shoulder at 222 nm (Ikeda et al. 1972).

After 8 h incubation period, fibrillar lysozyme (black) shows a loss of the native spectrum in Figure X (B) shows the structural change from native lysozyme to amyloid fibers where α-helix passes from 53.4% to 0%, all β-sheet change from 2.1% parallel an 11.8% antiparallel to 40.1% antiparallel and 0 parallel, the turns pass from 3.8% to 14.6% and others from 28.8% to 45.3%. These changes suggest a notable increase of antiparallel β-sheet and the loss of the other structures. This structure is more common at smaller scales and timescales and is the first to appear in many amyloid aggregation processes (Zanjani et al. 2020). When L-tyr is present, lysozyme conserves α-helix with 22.9% and has less change to β-sheet only 23.3%, turns changes to 13.3% and others to 40.6%. This result suggests that the final structure of the lysozyme when the L-tyr is present is an intermediate that is created for the interruption in behavior of the process from native to fiber. The increase of β-sheet could be responsible for the property of binding ThT and ANS. This is supported by the results obtained from ThT and ANS assays, where the same behavior was.

### Thermal denaturation

To elucidate the effect of L-tyr, we compared heat denaturation profiles under two conditions: lysozyme alone and lysozyme with L-tyr. This comparison provides insights into whether L-tyr acts as a stabilizer for the protein.

The thermal denaturation experiment was conducted over a 25–90 °C temperature range. To effectively analyze the influence of L-tyr, the data was divided into two distinct segments: the first from 25 to 50 °C and the second from 50 to 90 °C. This segmentation allows us to clearly observe the effects of L-tyr on protein stability throughout different phases of thermal stress.

**Fig. 5:**
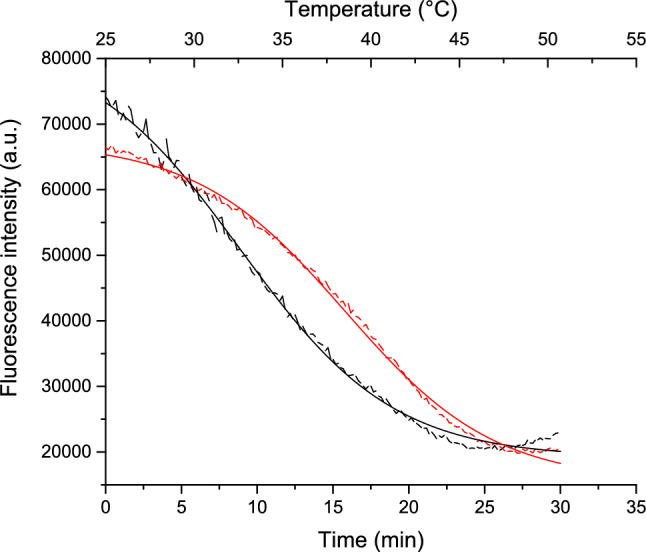
Heat denaturation of lysozyme monitored by tryptophan fluorescence. The figure displays the thermal denaturation profiles of lysozyme over a temperature range of 25–50 °C with tryptophan fluorescence intensity as the reporter. Data for lysozyme alone is represented by the black dotted line, while lysozyme in the presence of L-tyr is shown with a red dotted line. Continuous lines represent the best fit to the data according to Eq. 1

Figure 5 presents the results for the first segment of the thermal denaturation. This initial phase highlights changes in tryptophan fluorescence quenching, which is associated with alterations in the protein environment as temperature increases (Eisinger and Navon 1969). By analyzing these changes, we can assess how L-tyrosine affects the thermal stability and structural integrity of lysozyme.

As illustrated in Fig. 5, the black line represents the heat denaturation profile of lysozyme alone, while the red line shows the profile in the presence of L-tyr. The denaturation processes are similar, in the initial and final stages but diverge in the intermediate phase. The fluorescence intensity quenching observed suggests a progressive exposure of aromatic amino acids from an hydrophobic to a polar environment (Lefevre et al. 2007).

The heat denaturation data were analyzed using Eq. 1, as outlined in the Materials and Methods Sect. "Heat denaturation.". Two critical parameters were assessed: (1)x0, the midpoint of fluorescence intensity change, and (2) dx, the time constant.

1. Midpoint (x0): This parameter represents the time at which half of the fluorescence intensity change occurs, reflecting the midpoint of aromatic amino acid exposure. For lysozyme alone (black line), x 0 is 8.90 ± 0.17 min. In the presence of L-tyr (red line), x 0 shifts to 15.81 ± 0.09 min. This increase of 6.91 min suggests that L-tyr delays the exposure of tryptophan to the solvent, indicating that the compound stabilizes the protein during the initial denaturation phase.
2. Time constant (dx): This parameter indicates the rate of tryptophan quenching and solvent exposure. For lysozyme alone, dx is 5.04 ± 0.12 min, whereas, for lysozyme with L-tyr, is 4.93 ± 0.10 min. The similarity in dx values suggests that the overall rate of the denaturation process is comparable in both conditions. However, the increased dx value in the presence of L-tyr indicates that the initial denaturation phase is slower, while the latter phase proceeds at a similar rate to lysozyme alone.

These findings suggest that L-tyr stabilizes lysozyme during the early stages of denaturation but accelerates the process in the later stages. The interaction site of L-tyr may be crucial for modulating the denaturation process and the transition of lysozyme to amyloid fibers. Further 55–90 °C analysis revealed increased tryptophan fluorescence, potentially indicating an aggregation process. Light scattering experiments have demonstrated the formation of lower-order associated species between 50 and 70 °C, followed by rapid cooperativity into β-amyloid fibrils (Sharma et al. 2016).

**Fig. 6:**
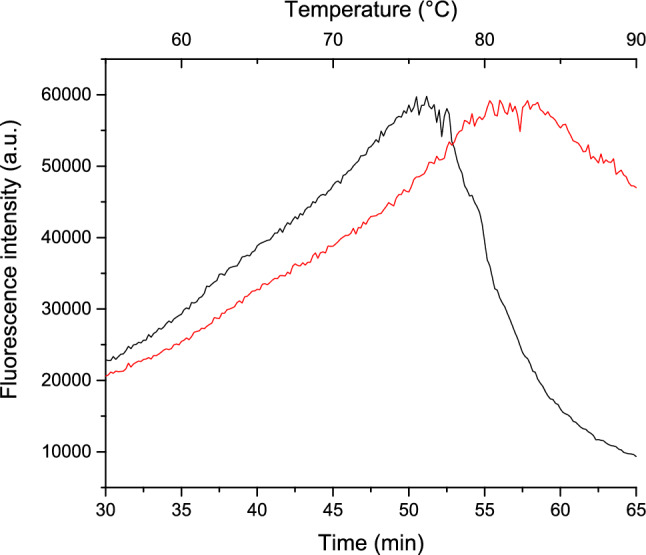
Heat denaturation of lysozyme (ranging from 50 to 90 °C) monitored by tryptophan fluorescence intensity. The black line represents lysozyme alone, while the red line corresponds to lysozyme in the presence of L-tyr

In the second phase of heat denaturation, the presence of L-tyr shifts the time and temperature required to reach the same maximum tryptophan fluorescence, as shown in Fig. 6. This increase in fluorescence signals structural changes and greater solvent exposure of tryptophan residues. In the first phase of lysozyme aggregation (30–50 min, 55–*ca*. 75 °C), the addition of L-tyr causes this range to shift to 30–55 min and 55 to *ca*. 80 °C, representing an increase of 5 min and 5 °C. This shift suggests that the stabilizing effect of L-tyr observed in the early stages of denaturation continues to be significant. However, the interaction between lysozyme and L-tyr intensifies, resulting in a more notable delay in aggregation.

During the second phase, the aggregation process for lysozyme occurs between 50 and 52 min at 75–77 °C, whereas in the presence of L-tyr these values are extended to 55–58 min and 80–83 °C. This demonstrates an additional delay of 3 min and 3 °C in the presence of L-tyr, further reinforcing its stabilizing effect. In the final phase, denaturation proceeds from 52 to 65 min at 77–90 °C for lysozyme alone, while for lysozyme in the presence of L-tyr, this process starts at 58 min and 83 °C. Unfortunately, the full range for the latter cannot be captured, as the endpoint is not recorded, limiting the comparison to the initiation of this phase.

These findings demonstrate that lysozyme in the presence of L-tyr undergoes more extended and slower heat denaturation phases than lysozyme alone. This rightward shift in Fig. 6 indicates that the presence of L-tyr enhances the thermal stability of lysozyme, delaying denaturation and aggregation.

The results of those experiments suggest that L-tyr exerts a moderate stabilizing effect during the initial stages of heat denaturation (25–55 °C), where the process occurs similarly in both conditions but along different pathways. However, in the latter stages (55–90 °C), the stabilizing effect of L-tyr becomes more pronounced, as evidenced by the delayed and slower denaturation. This implies that while L-tyr provides only mild stabilization during minor structural changes, it plays a more critical role in preventing major structural transitions in lysozyme, potentially inhibiting fibrillogenesis and the formation of amyloid fibers.

### Differential scanning calorimetry (DSC)

**Fig. 7:**
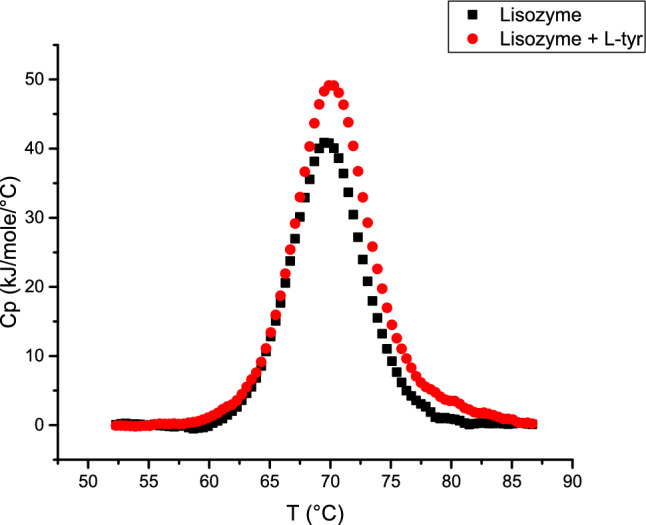
Differential scanning calorimetry (DSC) thermograms showing the effect of L-tyr on lysozyme stability. Black squares correspond to lysozyme alone, while red circles represent lysozyme in the presence of L-tyr

DSC experiments were conducted in the presence and absence of L-tyr, as illustrated in Fig. 7. The thermal profiles reveal distinct differences between the two conditions, providing insight into the stabilizing effect of L-tyr on lysozyme.

In both cases, a single endothermic peak typical of protein denaturation was observed (Cinelli et al. 2004). When comparing the parameters, the Tm values are nearly identical: 69.5 °C for lysozyme alone and 69.6 °C in the presence of L-tyr, with a negligible difference of 0.1 °C. However, for ΔH, the enthalpy change for lysozyme alone is 311.79 kJ ${\text{mol}}^{-1}$, while in the presence of L-tyr, it increases to 407.25 kJ ${\text{mol}}^{-1}$, a difference of 95.46 kJ ${\text{mol}}^{-1}$. This indicates that more heat energy is required to transition from the native to the denatured state when L-tyr is present.

Additionally, the heat capacity (Cp) values at Tm for lysozyme is 40.86 kJ ${^\circ \text{C}}^{-1}{\text{mol}}^{-1},$ while in the presence of L-tyr it raises to 49.14 kJ ${^\circ \text{C}}^{-1}{\text{mol}}^{-1}$, a difference of 8.28 kJ ${^\circ \text{C}}^{-1}{\text{mol}}^{-1}$. These results suggest that although the Tm remains unchanged, the energy required to reach this point is significantly higher when L-tyr is present. ΔH and Cp values can be used to indicate a protein’s conformational stability (Singh and Singh 2003; Johnson 2013).

Therefore, it is evident that L-tyr increases the energy required for denaturation, which supports the hypothesis from the previous section. L-tyr likely interacts with lysozyme as its structure begins to change, leading to increased protein stabilization.

### Denaturation by urea

**Fig. 8:**
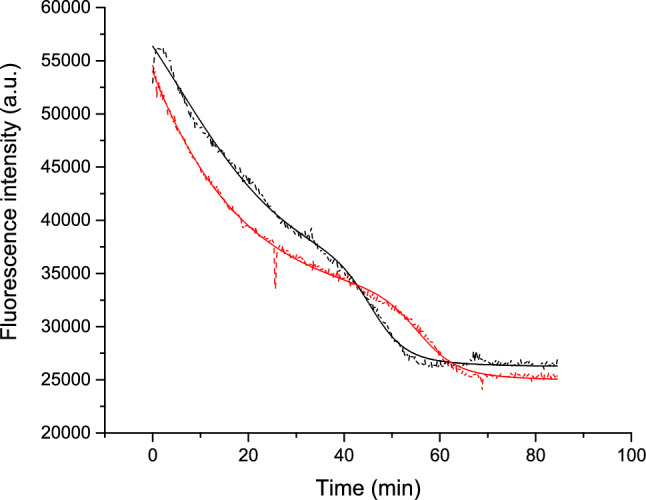
Urea-induced unfolding kinetics of lysozyme in the absence (black dotted line) and presence (red dotted line) of L-tyr at 6.5 M urea concentration. The solid lines represent the best data fits according to Eq. 2 (refer to Materials and Methods Sect. "Urea denaturation")

Urea-induced denaturation provides further insight into the stabilizing effect of L-tyr on lysozyme. As shown in Fig. 8, the presence of L-tyr significantly alters the typical denaturation behavior of the protein.

In this experiment, lysozyme’s intrinsic fluorescence was monitored for two hours under two conditions: lysozyme alone (black line) and lysozyme in the presence of L-tyr (red line). Urea disrupts the tertiary structure by interacting with hydrophilic residues, leading to a into a softer structure, a molten globule-like state, similar to the structural changes seen during thermal denaturation (Hédoux et al. 2006, 2010).

Several parameters derived from Eq. 2 (see Materials and Methods Sect. "Urea denaturation") were compared to quantify the effect. Two key time constants, h_1_, and h_2_, describe the unfolding kinetics; h_1_ corresponds to the initial denaturation phase, while h_2_ reflects the second phase. For h_1_, the values for lysozyme alone and in the presence of L-tyr were 0.03 ± 0.002 and 0.02 ± 0.002, respectively, indicating minimal differences in the initial phase. This suggests that urea initially targets hydrophilic residues similarly in both conditions (Hédoux et al. 2010). However, in the second phase, the h_2_ values were slightly different: 0.12 ± 0.008 for lysozyme alone and 0.10 ± 0.006 in the presence of L-tyr, suggesting a modest slowing of the denaturation process when L-tyr is present.

The midpoint of the second phase also shows a significant shift. Lysozyme alone reached this midpoint at 45.57 ± 0.16 min, whereas in the presence of L-tyr, it was delayed to 55.46 ± 0.16 min. This substantial delay indicates that L-tyr exerts a stabilizing effect on lysozyme, prolonging the denaturation process. The data suggest that L-tyr stabilizing influence persists across different types of denaturation, confirming that its protective effect is not dependent on the denaturation mechanism.

### Scanning electron microscopy (SEM)

Microscopic analysis is essential to verify the effect of L-tyr. This study focuses on three key cases: (1) native lysozyme, (2) amyloid fibers formed through fibrillogenesis, and 3) the fibrillogenesis process in the presence of L-tyr.

**Fig. 9:**
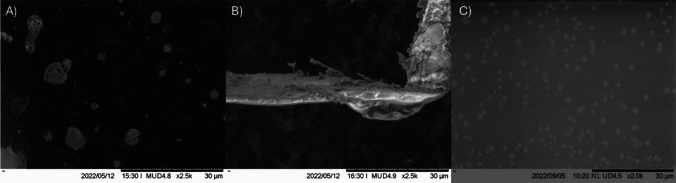
SEM images of lysozyme on different conditions. **A** Lysozyme natively folded. **B** Lysozyme amyloid fiber obtained with SE mode at 5 kV. **C** Lysozyme in the presence of L-tyr

SEM results, presented in Fig. 9, highlight the differences between the three experimental conditions. Panel A shows what the lysozyme looks like when correctly folded; in this case, the protein looks like a film. Panels B show amyloid fibers obtained with SE mode at 5 kV, shows better information on the sample morphology (Postek et al. 2016). In this image, larger fibers, consisting of smaller subunits, are evident, likely a consequence of the high protein concentration used (25 mg mL^−1^). As expected, when L-tyr is present, amyloid fibers are absent, as is shown in panel C. These images clearly demonstrate the inhibitory effect of L-tyr on amyloid fiber formation.

Figure 9C demonstrates that the inhibition of amyloid fibers by the presence of L-tyr leads to the formation of distinct structures resembling dots. These structures likely represent small aggregates with different conformations compared to the original lysozyme unlike amyloid fibers, these aggregates show a reduced binding affinity for ThT and ANS, suggesting they may represent an intermediate state between the native protein and fully-formed fibers; this is supported by the CD section where is demonstrated that the final structures are different. Given their form and characteristics, these dot-like structures could be spherulites. Spherulites have previously been observed in both egg white lysozyme (Heijna et al. 2007) and human lysozyme. In human lysozyme, spherulites have been reported when fibril formation is disrupted in the early stages by aggregation inhibitors such as arginine and benzyl alcohol (Sharma et al. 2016).

### Docking

**Fig. 10:**
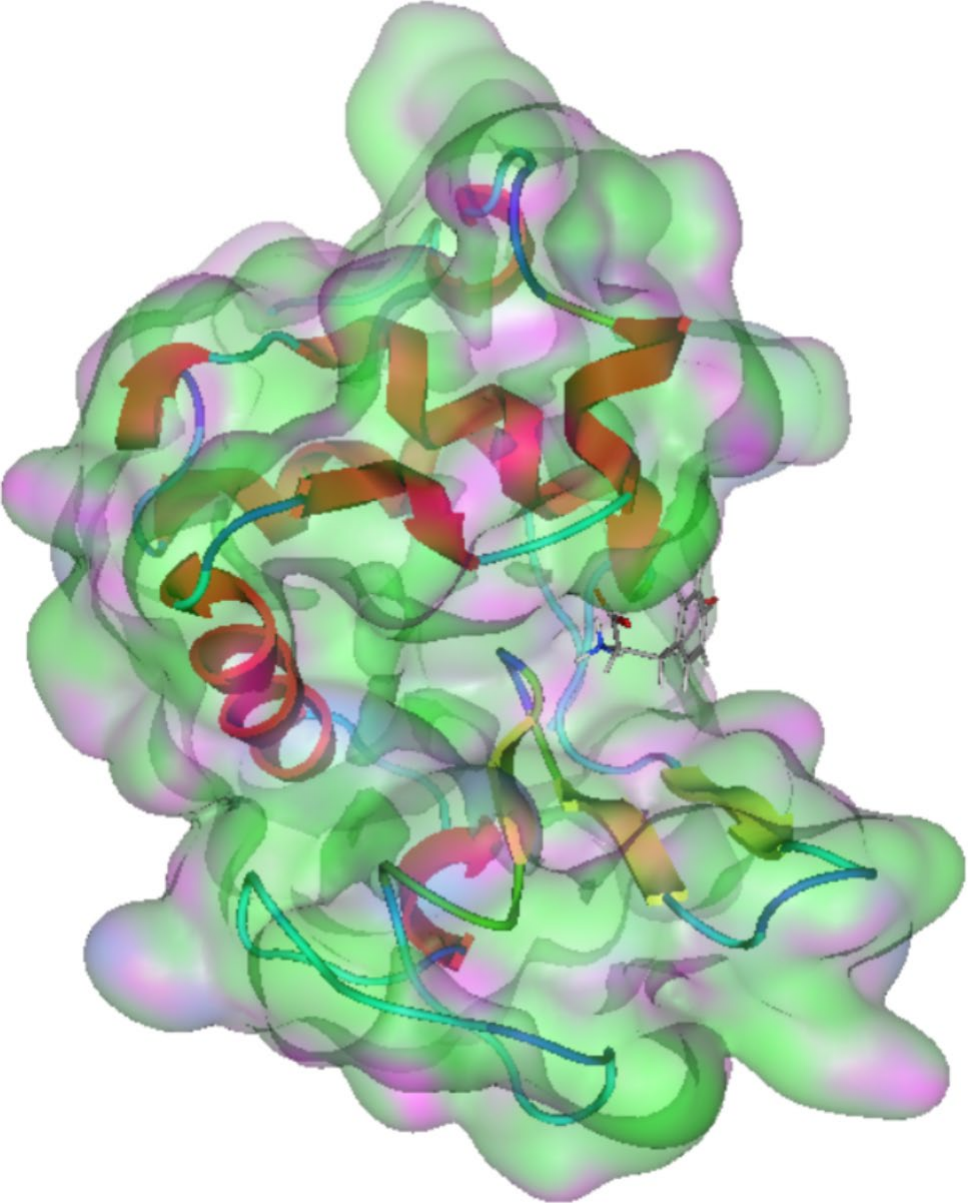
Human lysozyme docked with L-tyr, based on the PBD 3FE0 structure

**Fig. 11:**
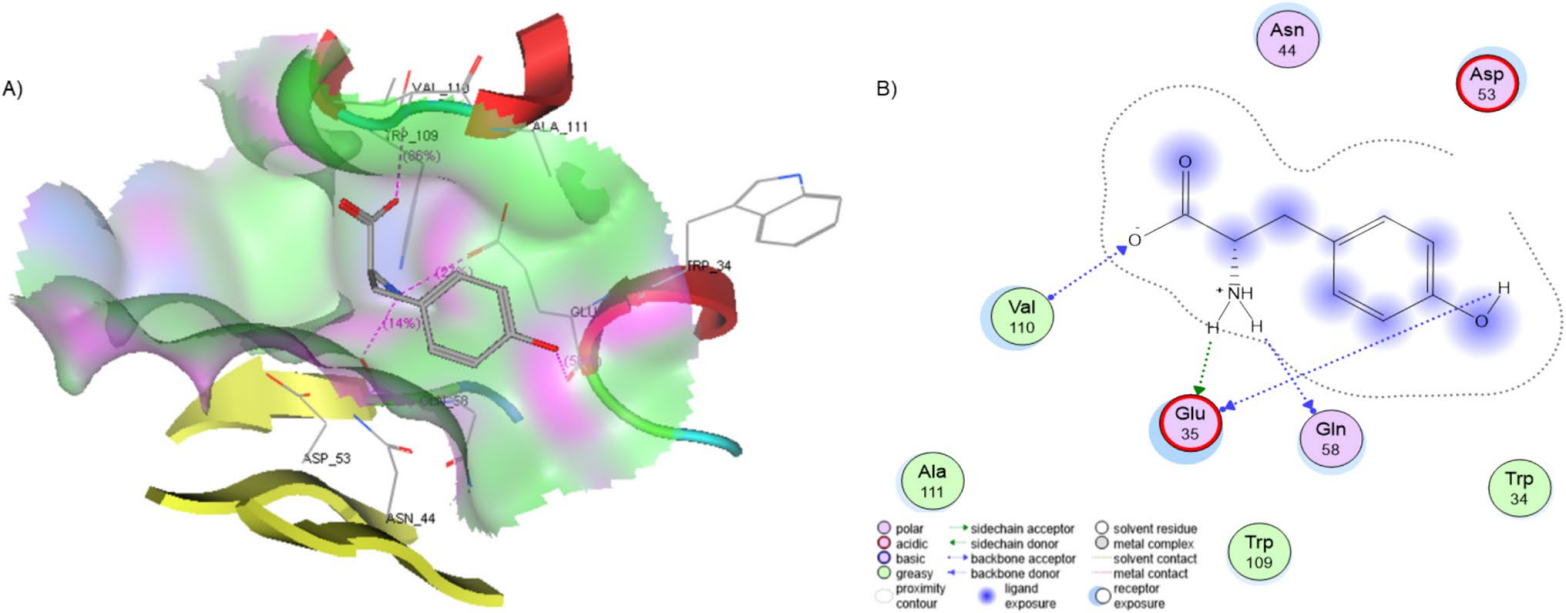
Interaction between lysozyme and L-tyr. **A** Binding of L-tyr at the interaction site within the lysozyme structure. **B** Interaction map detailing the specific molecular contacts between lysozyme and L-tyr, highlighting key residues involved in binding

Bioinformatics provides valuable insights into the underlying interaction mechanisms, offering more detailed information. Docking experiments, in particular, can identify the likely binding sites where a compound interacts with a protein (Chen 2015). In this study, PBD ID 3FE0 was used as a model for human lysozyme, and the compound structure was obtained from PubChem (Fig. 10). The docking process involved using all protein atoms to determine potential interaction sites. The results indicated that the compound primarily interacts with a specific protein region, as shown in Fig. 11. In this case, L-tyr was observed to partially enter the protein, localizing near the active site. This raises the question of whether these interactions mimic those between the protein and its natural substrate.

Lysozyme relies on two key amino acids for its enzymatic activity: Glu 35 and Asp 53 (Sophianopoulos 1969). Following the docking analysis, several important observations were made. First, the location and orientation of the L-tyr within the binding site are shown in Fig. 11A. In this position, L-tyr interacts with the alpha and beta domains of lysozyme, suggesting that this interaction could play a stabilizing role for the protein. This indicates that the interactions between L-tyr and lysozyme differ from those involving its natural substrate. As shown in Fig. 11B, the docking results reveal three key interactions between the two molecules, with only the interaction involving Glu 35 being shared with the natural substrate (Sophianopoulos 1969; Muraki et al. 1987).

The docking score is − 11.29 kcal/mol, and four hydrogen bonds are present. Glu35 forms two hydrogen bonds, one with the hydroxyl group of L-tyr with a distance of 1.58 Å, the second with the amino group and a distance of 1.42 Å, additional this group forms another hydrogen bond with Gln58 and 2.32 Å of distance. The Carboxy group forms a hydrogen bond with Val110 with a distance of 2.70 Å.

The docking analysis reveals a high-affinity binding site on lysozyme, which aligns with experimental evidence suggesting that L-tyrosine interacts with crucial structural amino acids. These residues act as stabilizing scaffolds, and the binding of L-tyr likely inhibits significant conformational changes, thereby preserving the protein's structural integrity. This interaction supports the hypothesis that L-tyr plays a key role in stabilizing lysozyme, preventing the destabilization that can lead to amyloid fibril formation.

The computational docking results complement the experimental findings and provide a detailed atomic-level insight into the protective mechanism of L-tyr. This structural view strengthens the understanding of how L-tyr inhibits lysozyme fibrillogenesis, highlighting its potential as a therapeutic strategy to counteract amyloid-related disorders. These findings emphasize the importance of integrating computational and experimental approaches to fully elucidate molecular mechanisms of protein stabilization.

## Conclusions

Our results demonstrate that lysozyme can form amyloid fibers under physiological conditions (pH 7.4, 37 ºC) and that L-tyrosine effectively inhibits this process at a 1:1 molar ratio. L-tyr acts as a potent stabilizer of the protein, as shown by denaturation experiments, where its presence significantly alters the denaturation profile of lysozyme. This indicates that L-tyrosine binding provides robust stabilization, helping the protein maintain structural integrity even in fluctuating environmental conditions. CD experiment demonstrated that the structure produced when the L-tyr is present is different from the native lysozyme and the fibers. This is confirmed by microscopy, revealing the formation of spherulites as intermediates in the inhibition process. Docking studies pinpoint a favorable binding site at the protein core, where L-tyr interacts with both lysozyme domains, a mechanism that likely drives the stabilization and inhibition observed in this study. These findings highlight L-tyrosine as a promising candidate for modulating protein stability and preventing amyloid fiber formation.

## Data Availability

No datasets were generated or analysed during the current study.
